# Diagnostic Difficulties in a Pediatric Insulinoma: A Case Report

**DOI:** 10.1097/MD.0000000000003045

**Published:** 2016-03-18

**Authors:** Ingrith Miron, Smaranda Diaconescu, Gabriel Aprodu, Ileana Ioniuc, Mihai Radu Diaconescu, Lucian Miron

**Affiliations:** From the Mother and Child Department (IM, SD, GA, II); Surgery Department (MRD); and Oncology Department (LM), “Gr. T. Popa” University of Medicine and Pharmacy, Iasi, Romania.

## Abstract

Insulinomas are functional neuroendocrine pancreatic tumors rarely encountered in pediatric pathology.

Insulinomas are usually solitary and sporadic, but may occur in association with multiple endocrine neoplasia type 1. Whipple's triad—hypoglycemia, simultaneous compatible adrenergic and/or neurological signs, and relief of symptoms upon the administration of glucose—remains the fundamental diagnostic tool.

We report a case of insulinoma in an 11-year-old boy with malnutrition and mild psychic retardation.

History revealed neuroglycopenic symptoms associated with hypoglycemia that returned to normal values after glucose intravenous infusion; before admission in our unit, the levels of circulating insulin, as well as the abdominal ultrasound and abdominal computed tomography scan, were reported within normal range. During hospitalization in our service, the glycemic curves showed recurring low values associated with low glycated hemoglobin, positive fasting test, and elevated C-peptide. The pancreatic ultrasound was inconclusive, but the magnetic resonance imaging revealed a high signal focal area with a diameter of 1 cm, located in the tail of pancreas. Conventional enucleation of the lesion prompted a spectacular normalization of glucose metabolism and the alleviation of the main clinical symptoms. The child had a favorable evolution in the clinical follow-up, presenting with weight gain and progressive remission to complete disappearance of most symptoms—except for the mental impairments.

Although in our case Whipple's triad was apparent from the beginning, the diagnosis was delayed due to the failure of conventional imaging methods in locating the tumor. Weight loss and mental impairment contributed to the diagnosis pitfalls.

Pediatricians should be aware of confusing and nonspecific symptoms, especially when children with insulinoma present mental or neurological retardation. Despite the existence of medical regimens, surgery remains the gold standard for the therapeutic approach to this condition.

## INTRODUCTION

Insulinomas are the most frequently encountered type of neuroendocrine pancreatic tumors, with a reported incidence of 1 to 4 cases/million persons/y.^1,2^ This kind of tumor can occur at any age—Das et al^3^ reported the case of a 4-month-old baby—with a slight predominance in females. Although most of the lesions are unique, approximately 10% of them are multiple and occur in association with multiple endocrine neoplasia type 1 (MEN1).^4^ Benign insulinomas are small tumors that usually measure around 1 to 2 cm. Although the classical Whipple triad—hypoglycemia, simultaneous compatible adrenergic and/or neurological symptoms, and prompt relief of these symptoms upon glucose intake—is pathognomonic, many pitfalls and errors may arise in insulinoma diagnosis. Standardized fasting test is mandatory, but the imaging work-up can sometimes be inconclusive for the objectification of masses that only measure a few millimeters. Although there are other recommended medical regimens, surgical removal remains the gold standard for this pathology.^5,6^

## CASE REPORT

**FIGURE 1:**
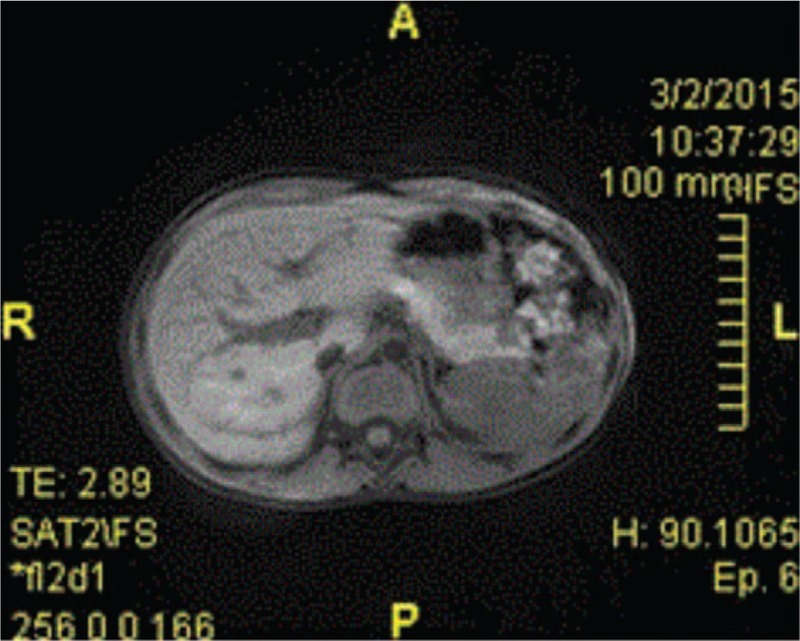
MRI: high signal focal area of 1 cm in diameter in the tail of the pancreas. MRI = magnetic resonance imaging.

An 11-year-old Caucasian boy was admitted into our unit with diffuse abdominal pain, cold sweats, confusion, tremor, and paresthesias. The patient came from a rural area, where he lived in a community with limited resources. His mother was 38, in apparent good health, and his father had died at the age of 50 (i.e., 3 years before the admission) from acute myocardial infarction. The patient had a 9-year-old sister with no significant medical history. The pregnancy was uneventful, with vaginal delivery at 39 weeks. The boy had no perinatal pathology, but since the age of 7 had presented numerous respiratory infections and uninvestigated mild psychic retardation. History revealed several episodes of faintness, myalgias, paraesthesias, and unexplained irritability; his mother also reported unusual hunger and tachycardia occurring mainly in the morning hours or after physical exercise. The symptoms started 5 months before the admission in the hospital service. He was admitted twice in a county hospital; although it was noticed that hypoglycemia (ranging from 14 to 38 mg/dL) would promptly return to normal values after glucose intravenous infusion, the levels of circulating insulin, as well as the abdominal ultrasound and abdominal computed tomography (CT) scan, were reported within normal range. The patient was finally diagnosed with “idiopathic intermittent” hypoglycemia. An informed consent was given by his mother upon admission; clinical examination revealed an impaired general condition, normal skin color, marked hypotrophy of stature, and weight (weight = 19.5 kg, height = 132 cm, body mass index = 17.7 kg/m^2^). The vital signs were as follows: temperature 98.4°F, heart rate 121 beats/min, respiratory rate 20 breaths/min, and blood pressure 96/67 mm Hg without any orthostatic changes. Physical examination revealed the following aspects: normal shaped head with no sign of trauma, intact extraocular muscles, equal and round pupils, reactive to light and accommodation, normal nostrils; well hydrated and lesion free oral cavity, with moist mucous membranes; lungs were clear to auscultation; regular heart rate and rhythm, with no murmurs; cold extremities; soft abdomen, presenting with no tenderness or distension, positive bowel sounds, no organic masses were noted. The patient described paresthesias, irritability, confusion, dizziness, and tremor; he was hypokinetic, presenting with uncoordinated movements of the extremities and slight limb spasticity. Neuropsychiatric examination ascertained the patient had a mild mental retardation. During hospitalization, the glycemic curves showed recurring values <50 mg/dL associated with low glycated hemoglobin (reported value: 3.90%; normal value: 4%–6%), a fasting insulinemia of 12.6 μU/mL (normal value <6 μU/mL) together with a postprandial insulinemia of 72.1 μU/mL, and elevated C-peptide level (reported value: 6.42 ng/mL; normal value <2 ng/mL). The urine sample was normal. Low serum glucose levels were closely monitored, and the patient received continuous intravenous serum glucose and electrolyte solutions. The pancreatic ultrasound was inconclusive, but the magnetic resonance imaging (MRI) revealed a high signal focal area with a diameter of 1 cm, located in the tail of pancreas (Figure 1).

**FIGURE 2:**
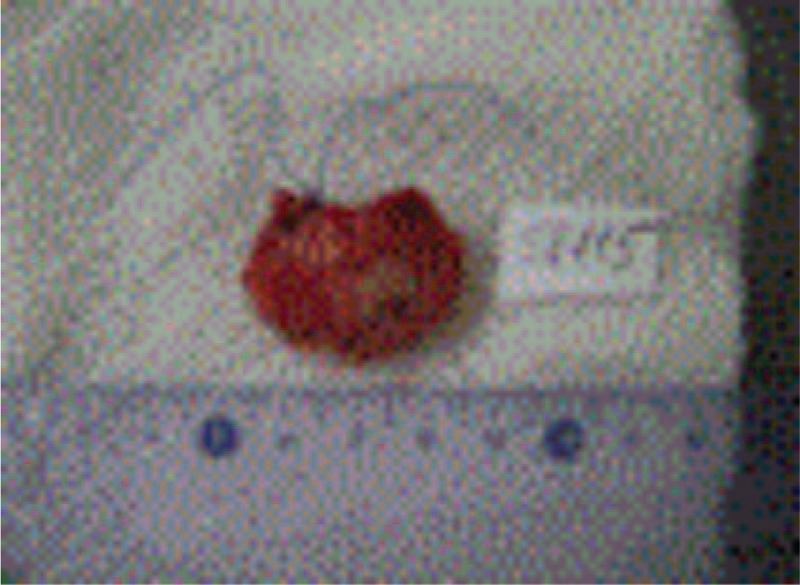
Insulinoma, operative piece.

**FIGURE 3:**
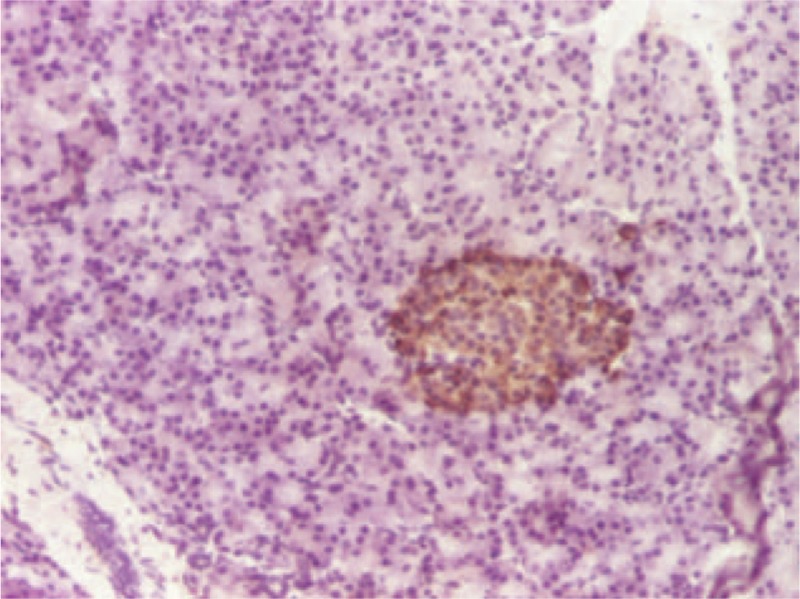
Immunohistochemistry positive for chromogranin A associated with the cytoplasmic neurosecretory granules of the tumor cells.

**FIGURE 4:**
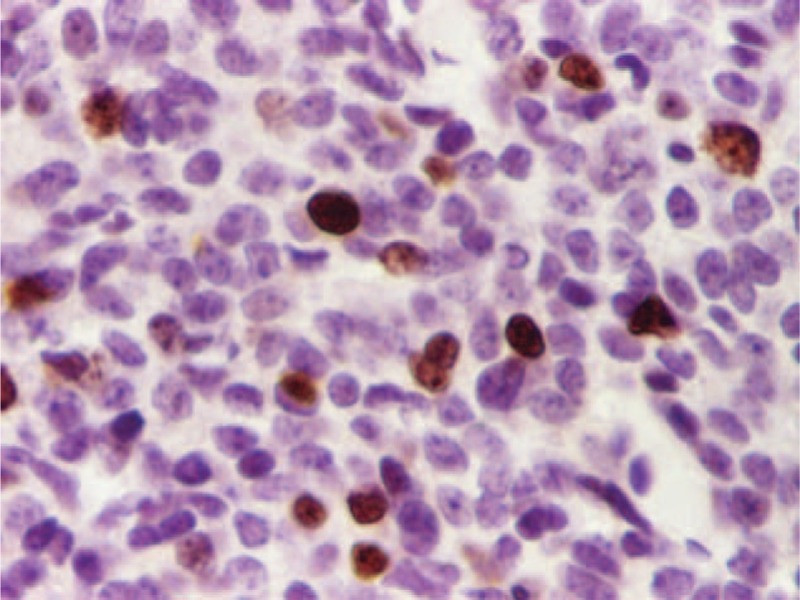
Nuclear Ki67 positivity (approximately 8%).

Considering the case history, the clinical, biochemical and imaging findings, and excluding other causes of hyperinsulinemic hypoglycemia—such as exogenous insulin administration or accidental ingestion of oral antidiabetic medicine—the patient was temporarily diagnosed with hypoglycemia-inducing caudal pancreatic tumor, which was a clear indication for surgical treatment. The patient underwent an open surgical procedure. Complete mobilization and careful exploration of the pancreas revealed a firm nodule located in the tail of the pancreas which was easily enucleated; the piece was routinely fixed in 4% formaldehyde and embedded in paraffin for pathological examination. The excised tumor measuring 1 cm in diameter was well-circumscribed, softer than the surrounding pancreatic parenchyma, and had a yellow-brownish cut surface that did not appear to be necrotic or cystic (Figure 2). Further immunohistochemical examination of the operative piece was positive for chromogranin A associated with the cytoplasmic neurosecretory granules of the tumor cells and approximately 8% of nuclear Ki67 positivity (Figures 3 and 4). The patient's immediate postoperative course was uneventful. Plasma insulin values promptly returned to normal, but transient hyperglycemia occurred and lasted for approximately 48 hours. However, no further episodes of hypoglycemia were recorded. The patient was discharged without hypoglycemic symptoms 8 days later. He currently has a favorable evolution in the clinical follow-up, presenting with weight gain and progressive remission to complete disappearance of most symptoms—except for the mental impairments.

## DISCUSSIONS

Insulinomas are a particular kind within the broad category of neuroendocrine pancreatic tumors by virtue of their incidence, pathogenic mechanisms, symptomatology and biology, as well as due to the diagnostic difficulties and pitfalls, the natural history, and last but not least, the therapeutic possibilities. These lesions are very rare, with around 1 to 4 new cases occurring per million persons per year, and particularly uncommon in children—the mid-20th century medical literature only gathering dozens of isolated or small series of cases.^7–11^ Although most insulinomas are solitary and sporadic—such as in our case —10% of them occur in connection with MEN1.^12–14^ Most insulinomas are benign, but up to 10% can be malignant.^14,15^ The clinical presentation of patients includes neuroglycopenic symptoms such as loss of consciousness, lethargy, confusion, dizziness, recurrent seizures, and coma. Other symptoms are related to the catecholamine response, such as palpitations, tachycardia, and hypertension, as well as hunger, weight gain, epigastric pain, or vomiting. Nonetheless, Whipple's triad remains the fundamental diagnostic tool.^16,17^ Although in our case the triad was apparent from the beginning, the diagnosis was delayed due to the failure of conventional imaging methods in locating the tumor. Unlike other cases reported by authors, this patient presented with weight loss.^18^ Also, the mental impairment could be attributed to the frequent hypoglycemic episodes and the psychological stress caused by the death of the patient's father, as well as to the lack of global stimulation due to poor education associated with a low socioeconomic status. Other authors reported diagnostic difficulties associated with confusing neurologic symptoms, such as recurrent seizures, that were diagnosed as drug-refractory epilepsy.^16–18^ Childhood insulinoma should not be confused with pancreatic nesidioblastosis or persistent hyperinsulinemic hypoglycemia of infancy (PHHI), which has a higher incidence in Saudi children and Ashkenazi Jewish populations, with a focal or diffuse hyperfunction of the β islet cells.^19^ PHHI has a very early onset (hours/days after the birth), presenting with persistent hypoglycemia and hyperinsulinism, and sometimes even leading to sudden death. The biological assessment of insulinomas includes glucose levels quickly dropping <40 mg/dL and C-peptide levels exceeding 0.6 μg/mL, as well as high levels of circulating insulin and proinsulin. The increase of serum immunoreactive insulin levels in response to secretin is significantly lower than in normal persons.^20^ A combination of gadolinium MRI, triple-phase CT, octreotide scanning, selective intra-arterial calcium stimulation, and particularly endoscopic and intraoperative ultrasound may actually detect almost all lesions.^21^ The initial treatment of childhood insulinoma is dietary and focused on preventing hypoglycemia. Diazoxide suppresses insulin secretion and enhances glycogenolysis, but has severe adverse effects including sodium retention, congestive heart failure, or hirsutism. The administration of somatostatin is frequently unsuccessful because unlike other neuroendocrine tumors many insulinomas do not express the necessary somatostatin receptors. Octreotide and lanreotide maintain insulinemia within reasonable limits. Systemic chemotherapy, including combination of streptozotocin and doxorubicin associated with chemoembolization, peptide-receptor radionuclide therapy, radiofrequency ablation, or cryotherapy, may lead to temporary palliation of symptoms and may inhibit tumor growth.^22,23^ However, surgery is the only radical method of treatment, whether by enucleation or limited distal, central or even cephalic resection (sometimes with laparoscopic approach) in order to ensure stable healing.^24,25^ Total pancreatectomy and lymphadenectomy, if necessary, is reserved for extremely rare malignant cases.^26^

## CONCLUSIONS

Insulinomas are the most frequent neuroendocrine tumors of the pancreas, yet with an extremely rare incidence during childhood. As with most other neuroendocrine tumors, insulinomas are often difficult to diagnose due to the unpredictable presentation of its symptoms and involve numerous biochemical and imaging tests to document the reality and topography of endogenous hyperinsulinemic hypoglycemia. Pediatricians should be aware of confusing and nonspecific symptoms, especially when the patient presents with mental or neurological retardation. Surgical removal is the mainstay of treatment for these tumors.
